# Longitudinal and Bidirectional Relationships Between Sleep, Emotional Wellbeing and Weight Status in Childhood and Adolescence: Growing Up in Scotland Cohort Analysis

**DOI:** 10.1111/jsr.70239

**Published:** 2025-11-14

**Authors:** Emma Louise Gale, Joanne Elizabeth Cecil, Andrew James Williams

**Affiliations:** ^1^ School of Medicine University of St Andrews St Andrews UK; ^2^ School of Health in Social Sciences University of Edinburgh Edinburgh UK

**Keywords:** body mass index, longitudinal, mental health, paediatric, puberty, secondary data analyses

## Abstract

Poor sleep and emotional wellbeing, which often decline during puberty, are associated with declines in metabolic health and are key determinants of childhood obesity. The aim of the study was to explore bidirectional associations between sleep, emotional wellbeing and obesity from ages 8 to 14 using the Growing Up in Scotland cohort. Secondary analyses were conducted on data from sweeps 7–10 (ages 8–14). Sleep duration was caregiver‐reported at age 8 and self‐reported at 14. Obesity was assessed using BMI percentiles (BMIp), derived from objective height and weight at ages 8, 10, 12 and 14. Emotional wellbeing was assessed using the Strengths and Difficulties Questionnaire caregiver‐report at 10 and 12 years, and self‐report at 14 years. Pearson correlations and adjusted regressions examined associations between sleep, BMIp and emotional wellbeing from ages 8 to 14. Analyses included 4157 participants (50.2% male). Shorter sleep at 8 years was associated with higher BMIp at 12 years (*β* = −0.172, CI (95%) = −0.335, −0.116, *p* < 0.001) and poorer emotional wellbeing at 10 (*β* = −0.120, CI (95%) = −0.179, −0.034, *p* < 0.001) and 12 years (*β* = −0.101, CI (95%) = −0.164, −0.026, *p* < 0.001). Poorer emotional wellbeing at 10 (*β* = −0.116, CI (95%) = −0.232, −0.093, *p* < 0.001) and 12 years (*β* = −0.194, CI (95%) = −0.328, −0.174, *p* < 0.001) predicted shorter sleep at 14 years. Higher BMIp at 8 years was associated with shorter sleep at 14 years (*β* = −0.182, CI (95%) = −0.359, −0.111, *p* < 0.001). Poorer emotional wellbeing at 10 years was associated with higher BMIp at 14 years (*β* = 0.142, CI (95%) = 0.074, 0.629, *p* = 0.13). Bidirectional associations emerged between short sleep and emotional wellbeing and higher BMIp. Poor emotional wellbeing was associated with subsequent obesity; but not bidirectionally. These interactions should inform the design of health interventions for 8–14‐year‐olds.

## 1 Background

Extensive research has demonstrated how poor sleep, and mental health can adversely affect metabolic health during childhood and adolescence. Mechanistically, insufficient sleep can disrupt hormonal regulation, particularly affecting appetite‐related hormones such as leptin and ghrelin (Hart et al. 2013; Taheri et al. 2004), and stress‐response hormones like cortisol (Dockray et al. 2009). These disruptions contribute to increased hunger, reduced satiety (Hogenkamp et al. 2013) and elevated stress levels (Hirotsu et al. 2015) which can drive overeating and unhealthy behaviours (Saleh‐Ghadimi et al. 2019; Michels et al. 2012). Poor sleep also promotes systemic inflammation (Song et al. 2022) and insulin resistance (St‐Onge et al. 2023), compounding metabolic dysfunction and increasing the risk of obesity (Thota et al. 2017; Sesé et al. 2012). Similarly, poor mental health, characterised by emotional distress such as anxiety and depression, has been associated with alterations in neuroendocrine function and inflammation (Song et al. 2022), which may also predispose individuals to metabolic disorders (Kim et al. 2018; Nousen et al. 2014). Suggesting, the relationships between sleep, obesity and mental wellbeing are likely complex and bidirectional.

Research on developmental health trajectories has found that distinct patterns of poor sleep, declining mental health and increasing obesity prevalence emerge during late childhood and early adolescence (Gale, Cecil, and Williams 2024). Sleep duration often begins to decline between 8 and 14 years of age, emotional symptoms increase between 10 and 14 years, and obesity rates climb from around age 10, all coinciding with the onset of puberty (Gale, Cecil, and Williams 2024). Pubertal changes, including hormonal shifts (e.g., melatonin suppression, increases in sex and growth hormones) (Blakemore et al. 2010), physical growth (Saenger 2004) and psychosocial pressures (Porter et al. 2010; Tremblay and Frigon 2005), contribute to the emergence of health challenges. Hormonal changes disrupt sleep–wake cycles (Kirshenbaum et al. 2023; Lucien et al. 2021), while emotional changes can increase stress‐related eating behaviours (Calcaterra et al. 2021; Harris et al. 2015) and reduce physical activity (Gallant et al. 2023), amplifying the risk of obesity (Roemmich and Rogol 1999). This developmental window is therefore a critical period for intervention (Dorn et al. 2019).

While systematic reviews have explored the relationships between mental wellbeing and obesity (Amiri and Behnezhad 2019; Mannan et al. 2016), sleep and obesity (Gale, Williams, and Cecil 2024; Li et al. 2017), as well as poor mental wellbeing as a shared determinant of sleep and obesity (Gale et al. 2025), no studies have yet examined the combined interaction of all three factors longitudinally during the transition from childhood to adolescence. The aim of this research was to explore the longitudinal and bidirectional relationships between sleep duration, emotional wellbeing and obesity during the developmental transition in childhood from 8 to 14 years, using the Growing Up in Scotland (GUS) birth cohort 1 dataset (Scottish Centre for Social Research SG 2006). These secondary analyses will identify key periods when these issues emerge, map their cascading effects through late childhood and adolescence and highlight opportunities for effective intervention. The findings will inform holistic, targeted approaches to address these interconnected challenges and optimise outcomes during this critical developmental stage.

## 2 Materials and Methods

### 2.1 Ethics Statement

Ethical approval for this study was granted by the School of Medicine Ethics Committee, acting on behalf of the University Teaching and Research Ethics Committee (UTREC) at the University of St Andrews (Approval Code: MD16516, approved 29 September 2022; UK Data Service Special Licence dataset SN 5760, Project 226100). The data were collected with written consent, de‐identified and anonymised prior to us receiving them.

### 2.2 Study Design, Recruitment and Data Collection

The GUS study is an ongoing longitudinal research project that investigates children's health, development and behaviour from birth to 14 years in Scotland (a high‐income country with a temperament climate). Data collection began in 2005/06 when participants were 10 months old and continued incrementally over time, with the most recent data collected at age 16 (Growing Up in Scotland 2025). Children and their caregivers were recruited using Child Benefit records supplied by HM Revenue and Customs (Bradshaw et al. 2005). The initial cohort consisted of 5271 children and their caregivers across Scotland (Bradshaw et al. 2005) that met the following criteria (i) born between June 2004 and May 2005, and (ii) living within a Scottish local authority. To increase the sample size, an additional 502 participants were recruited using the following criteria: (i) mothers aged 16–24 at the time of the child's birth and/or (ii) residence in the 15% most deprived areas as identified by the 2016 Scottish Index of Multiple Deprivation (Knudsen et al. 2020b).

At each stage of data collection, a variety of methods were used, including physical measurements and interviews with key individuals such as the main caregiver, partner, teacher or child (Knudsen et al. 2020a; Bradshaw et al. 2018, 2015, 2013, 2011, 2010, 2009, 2007, 2006; Corbett et al. 2008). The focus of these measures evolved over time, with early sweeps prioritising birth outcomes and developmental milestones, and later sweeps incorporating age‐relevant topics such as behaviour, wellbeing, diet, physical activity, social interactions and experiences at school and home (Knudsen et al. 2020a; Bradshaw et al. 2018, 2015, 2013, 2011, 2010, 2009, 2007, 2006; Corbett et al. 2008). Data for sweeps 1–9 were gathered through in‐home interviews conducted by trained professionals using laptops, with a primary emphasis on quantitative closed‐ended questions (Bradshaw et al. 2018, 2015, 2013, 2011, 2010, 2009, 2007, 2006; Corbett et al. 2008). For sweep 10, data collection methods shifted in response to the COVID‐19 pandemic, moving from in‐person interviews (conducted from January to March 2020) to remote options, including telephone and online surveys (conducted from March to December 2020) (Knudsen et al. 2020b). The main caregiver, most often the child's birth mother, completed a detailed questionnaire during each sweep. These interviews, lasting between 60 and 80 min, gathered socio‐environmental and demographic information about the caregiver, the household and the child (Knudsen et al. 2020a; Bradshaw et al. 2018, 2015, 2013, 2011, 2010, 2009, 2007, 2006; Corbett et al. 2008).

### 2.3 Measures

#### 2.3.1 Child‐Specific Demographics

In every sweep (sweeps 1–10), the child's age (years) and sex were to be reported by the main carer (Knudsen et al. 2020a; Bradshaw et al. 2018, 2015, 2013, 2011, 2010, 2009, 2007, 2006; Corbett et al. 2008). Puberty status was child‐reported in sweep 10 using male‐specific and female‐specific body development questions (Knudsen et al. 2020a). For example, male‐specific questions included ‘Have you noticed a deepening in your voice?’ and ‘Has hair begun to grow on your face?’ If the child answered yes to either of those questions, they were then asked at what age they noticed the change (Knudsen et al. 2020a). The female‐specific questions included ‘have you ever menstruated (had your period)?’ If the child answered yes, they were then asked at what age the first menstruation occurred (Knudsen et al. 2020a).

#### 2.3.2 Caregiver‐Specific Demographics

The main carer and partner's ethnicity and education level were recorded in sweeps 2–10 reported by the main carer (Knudsen et al. 2020a; Bradshaw et al. 2018, 2015, 2013, 2011, 2010, 2009, 2007; Corbett et al. 2008). Socioeconomic status (SES) was assessed in sweeps 1–10, using the National Statistics Socio‐Economic Classification (NS‐SEC) (Rose and O'Reilly 1997), which considered the main carer's or partner's work status and profession. Each category was assigned a number: (1) ‘managerial and professional’, (2) ‘intermediate’, (3) ‘small employers and own account workers’, (4) ‘lower supervisory and technical’, (5) ‘semi‐routine’ and ‘routine occupations’ and (6) ‘never worked’ (Knudsen et al. 2020a; Bradshaw et al. 2018, 2015, 2013, 2011, 2010, 2009, 2007, 2006; Corbett et al. 2008). The SES of the main carer, partner and household NS‐SEC were recorded (Knudsen et al. 2020a; Bradshaw et al. 2018, 2015, 2013, 2011, 2010, 2009, 2007, 2006; Corbett et al. 2008). A household's SES status trajectory was derived for cases that had seven sweeps of NS‐SEC data. Differences between the mean and standard deviation of each valid trajectory, were based on non‐missing observations. If the standard deviation of the trajectory was less than 0.4, the case was labelled as a ‘static’. Where the standard deviations were above 0.4, the case was classified as overall ‘increasing’ or overall ‘decreasing’.

#### 2.3.3 Anthropometrics

The child's BMIp was derived by weight (kg) divided by height^2^ (m^2^) measured by trained professionals in sweeps 7–10 (Knudsen et al. 2020a; Bradshaw et al. 2018, 2015, 2013). Standard cut‐offs (International Obesity Task Force) were used to classify into five weight status groups using the Centers for Disease Control percentiles (Cole and Lobstein 2012): underweight (< 5th percentile), healthy weight (5th ≤ to < 85th percentile), overweight (85th ≤to < 95th percentile), obese (95th ≤ to < 98th percentile) and morbidly obese (≥ 98th percentile) (Cole and Lobstein 2012).

#### 2.3.4 Sleep Duration

Sleep duration was assessed subjectively through caregiver reports at age 8 and child self‐reports at age 14 years. At the child's age of 8 years, the main caregiver was asked, ‘Roughly how many hours of sleep does ^ChildName typically get in a 24‐h period, including any daytime naps?’ At the child's age of 14 years, the child participant was asked, ‘How many hours of sleep do you get on a school night and on a non‐school night?’ (Knudsen et al. 2020a; Bradshaw et al. 2013). Sleep duration data was not collected at age 10 or 12 years.

#### 2.3.5 Emotional Wellbeing

Emotional wellbeing was assessed using the emotional symptoms subscale from The Strengths and Difficulties Questionnaire (SDQ), with a higher score indicating emotional symptom difficulties (Goodman 1997). The SDQ was main‐carer reported in sweep 8 (Bradshaw et al. 2015) and child‐reported in sweep 10 (Knudsen et al. 2020a).

### 2.4 Data Analyses

Data were imported and analysed using the Statistical Package for Social Sciences (SPSS 28 Statistics). Missing data were assessed using the Little's Missing Completely at Random (MCAR) test, which statistically estimates the likelihood that missing values within a dataset are missing completely at random (Little 1988). Frequency descriptives, Pearson correlation and regression analyses were used. Tests were two‐tailed tests and used *α* = 0.05. Regression analyses were adjusted for child age, sex, pubertal status and emotional symptoms.

## 3 Results

### 3.1 Missing Data

This Little's MCAR test (*χ* ^2^ = 1786.975, df = 1401, *p* = 0.001) indicated that missing data were not missing at random (Little 1988). Depending on the statistical test, missing values were handled using listwise deletion for descriptives and frequencies or pairwise deletion for Pearson correlations and regression analyses. Findings were interpreted based on the available sample.

### 3.2 Sample Characteristics

*Demographic*s: The sample included 4157 participants, 50.2% male (Table 1). Full demographic details are presented in Table 1.

**TABLE 1:** Sample descriptive (*N* = 4157).

		*N*	%	Mean	SD
Child gender	Male	2085	50.2		
	Female	2072	49.8		
Caregiver ethnicity	White	3044	96.4		
	Other ethnic background	113	3.6		
Caregiver BMI (kg/m^2^)		3081		26.9	5.5
Caregiver education level	Under 16 years	84	15.4		
	16–18 years	363	66.5		
	19–24 years	82	15.0		
	25 years or older	14	2.6		
	Still in full‐time education	3	0.5		
Caregiver NS‐SEC (SES)	Managerial and professional occupations	1818	57.6		
	Intermediate occupations	453	14.3		
	Small employers/self‐employed	221	7.0		
	Lower supervisory and technical occupations	240	7.6		
	Semi‐routine and routine occupations	394	12.5		
	Never worked	31	1.0		
Caregiver NS‐SEC (SES) trajectory	Static	2788	88.3		
	Decrease	189	6.0		
	Increase	180	5.7		
Child sleep duration (h)	8 years	2674		10.4	1
	14 years	2351		7.9	1.1
Child BMIp	8 years	2636		0.7	0.3
	10 years	2554		0.7	0.3
	12 years	2597		0.7	0.3
	14 years	2056		0.6	0.3
Child emotional wellbeing (/10)	10 years	2589		1.6	1.8
	12 years	2598		1.8	2.1
	14 years	2342		1.8	2.1

Abbreviations: BMI, body mass index; *h, hours; N*, number; NS‐SEC, National Statistics Socio‐Economic Classification; SD, standard deviation; SES, socioeconomic status; SN, school night; WE, weekend.

*Sleep*: Mean sleep duration decreased with age, averaging 10.4 ± 1.0 h at age 8, and 7.9 ± 1.1 h at age 14 (Table 1).

*Obesity*: Mean BMIp was 0.7 ± 0.3 at 8, 10 and 12 years and was 0.6 ± 0.3 at 14 years (Table 1).

*Emotional wellbeing*: Mean emotional wellbeing score (/10) at 10 years was 1.6 ± 1.8.

### 3.3 Longitudinal Relationship Between Sleep, Obesity and Emotional Wellbeing

#### 3.3.1 Unadjusted Analyses

At age 14, girls demonstrated significantly shorter sleep duration, higher BMIp and poorer emotional wellbeing than boys (Table 2). Caregiver ethnicity was not significantly associated with sleep duration, BMIp, or emotional wellbeing (Table 2). A higher caregiver BMIp was strongly linked to a higher child BMIp at ages 10, 12 and 14, as well as poorer emotional wellbeing in children at ages 12 and 14 (Table 2).

**TABLE 2:** Pearson correlations of child and caregiver demographics, child sleep duration, child BMIp and child emotional wellbeing.

	1	2	3	4	5	6	7	8	9	10	11
Child gender	—										
2 Caregiver ethnicity	−0.013	—									
3 Caregiver BMI	0.013	0.006	—								
4 Child sleep duration (8 years)	**0.088**	**−0.047**	**−0.053**	—							
5 Child sleep duration (14 years)	**−0.107**	−0.002	**−0.053**	**0.123**	—						
6 Child BMIp (10 years)	**0.044**	**−0.048**	**0.237**	**−0.060**	−0.028	—					
7 Child BMIp (12 years)	**0.050**	−0.018	**0.252**	**−0.065**	**−0.050**	**0.851**	—				
8 Child BMIp (14 years)	**0.100**	−0.035	**0.242**	**−0.047**	**−0.047**	**0.762**	**0.854**	—			
9 Child emotional wellbeing (10 years)	0.024	−0.009	**0.077**	**−0.083**	**−0.105**	0.031	**0.042**	**0.064**	—		
10 Child emotional wellbeing (12 years)	0.010	0.017	**0.118**	**−0.105**	**−0.138**	0.034	**0.059**	0.038	**0.626**	—	
11 Child emotional wellbeing (14 years)	**0.121**	−0.004	**0.142**	**−0.054**	**−0.226**	0.044	**0.063**	0.040	**0.528**	**0.637**	—

*Note:* Bold—*p* > 0.01. Meaningful association—*r* > 0.1 and *r* < −0.01. Blue—negative association; yellow—positive association. A colour gradient was used to represent the strength and direction of associations, with solid colours indicating stronger associations and lighter shades indicating weaker ones.

Abbreviations: BMI, body mass index; BMIp, body mass index percentile.

Shorter child sleep duration at age 8 was associated with poorer child emotional wellbeing at age 12, while shorter child sleep duration at age 14 was associated with poorer child emotional wellbeing at ages 10, 12 and 14 (Table 2). Weak associations were observed between shorter child sleep duration and higher child BMIp at ages 10, 12 and 14, as well as between shorter child sleep and poorer child emotional wellbeing at ages 10 and 14 (Table 2). Additionally, a higher child BMIp at age 10 was weakly associated with poorer child emotional wellbeing at ages 10, 12 and 14, while a higher child BMIp at age 14 was weakly associated with poorer child emotional wellbeing at ages 12 and 14 (Table 2).

#### 3.3.2 Adjusted Analyses

Regression analyses were conducted to evaluate the bidirectional associations between sleep duration, obesity and emotional wellbeing from ages 8 to 14, adjusting for child gender, child pubertal status, caregiver ethnicity, caregiver SES trajectory and caregiver education level (Table 3). Significant longitudinal associations were identified, including poorer child emotional wellbeing at age 10 being significantly associated with a higher child BMIp at age 14, and shorter child sleep duration at age 8 being significantly correlated with a higher child BMIp at age 12, and poorer emotional wellbeing at ages 10 and 12. Furthermore, a higher BMIp at 8 years old was significantly associated with a shorter sleep duration at 14 years old. Poorer child emotional wellbeing at age 10 was significantly correlated with shorter child sleep duration at age 14, as was poorer child emotional wellbeing at age 12 (Table 3).

**TABLE 3:** Regression analyses (unadjusted and adjusted) of the relationship between sleep duration at 8 and 14 years, emotional wellbeing at 10, 12 and 14 years and body mass index percentile at 10, 12 and 14 years.

	Unadjusted				Adjusted			
	*β*	*t*	CI (95%)	*p*	*β*	*t*	CI (95%)	*p*
*Body mass index percentile and emotional wellbeing*								
BMIp (10 years) → EWB (12 years)	0.024	1.201	−0.002, 0.009	0.230	0.025	1.233	−0.002, 0.009	0.218
BMIp (10 years) → EWB (14 years)	0.026	1.229	−0.002, 0.009	0.219	0.022	1.024	−0.003, 0.009	0.306
BMIp (12 years) → EWB (14 years)	0.045	2.122	0.000, 0.012	0.034	0.040	1.870	0.000, 0.012	0.062
EWB (10 years) → BMIp (12 years)	0.034	1711	−0.031, 0.461	0.087	0.033	1.636	−0.041, 0.452	0.102
EWB (10 years) → BMIp (14 years)	**0.141**	**2.665**	**0.099, 0.652**	**0.008**	**0.142**	**2.482**	**0.074, 0.629**	**0.013**
EWB (12 years) → BMIp (14 years)	0.038	1.669	−0.047, 0.577	0.095	0.040	1.737	−0.036, 0.592	0.083
*Body mass index percentile and sleep duration*								
BMIp (8 years) → SD (14 years)	−0.088	−3.110	−0.312, −0.056	0.003	**−0.182**	**−3.622**	**−0.359, −0.111**	**< 0.001**
BMIp (10 years) → SD (14 years)	−0.021	−0.972	−0.017, 0.006	0.331	−0.018	−0.828	−0.016, 0.006	0.408
BMIp (12 years) → SD (14 years)	−0.043	−2.040	−0.023, 0.000	0.041	−0.039	−1.832	−0.022, 0.001	0.067
SD (8 years) → BMIp (10 years)	−0.062	−3.101	−0.358, −0.081	0.002	−0.068	−3.405	−0.379, −0.102	< 0.001
SD (8 years) → BMIp (12 years)	−0.088	−3.210	−0.313, −0.045	0.004	**−0.172**	**−3.526**	**−0.335, −0.116**	**< 0.001**
SD (8 years) → BMIp (14 years)	−0.056	−2.081	−0.323, −0.010	0.038	−0.065	−2.573	−0.399, −0.020	0.010
*Sleep duration and emotional wellbeing*								
SD (8 years) → EWB (10 years)	−0.095	−4.349	−0.067, −0.024	< 0.001	**−0.120**	**−4.559**	**−0.179, −0.034**	**< 0.001**
SD (8 years) → EWB (12 years)	−0.090	−4.596	−0.064, −0.026	< 0.001	**−0.101**	**−4.622**	**−0.164, −0.026**	**< 0.001**
SD (8 years) → EWB (14 years)	−0.058	−2.771	−0.049, −0.008	0.006	−0.070	−3.365	−0.055, −0.014	< 0.001
EWB (10 years) → SD (14 years)	−0.099	−4.699	−0.236, −0.097	< 0.001	**−0.116**	**−4.566**	**−0.232, −0.093**	**< 0.001**
EWB (12 years) → SD (14 years)	**−0.134**	**−6.400**	**−0.326, −0.113**	**< 0.001**	**−0.194**	**−6.392**	**−0.328, −0.174**	**< 0.001**

*Note:* Bold *β* > 0.1 and *β* < −0.1. Adjusted analyses were controlled for child gender, child pubertal status (at 14 years), caregiver ethnicity, caregiver educational level, caregiver BMI and caregiver SES trajectory.

Abbreviations: BMIp, body mass index percentile; CI, confidence interval; EWB, emotional wellbeing; SD, sleep duration.

## 4 Discussion

### 4.1 Overall Findings

**FIGURE 1:**
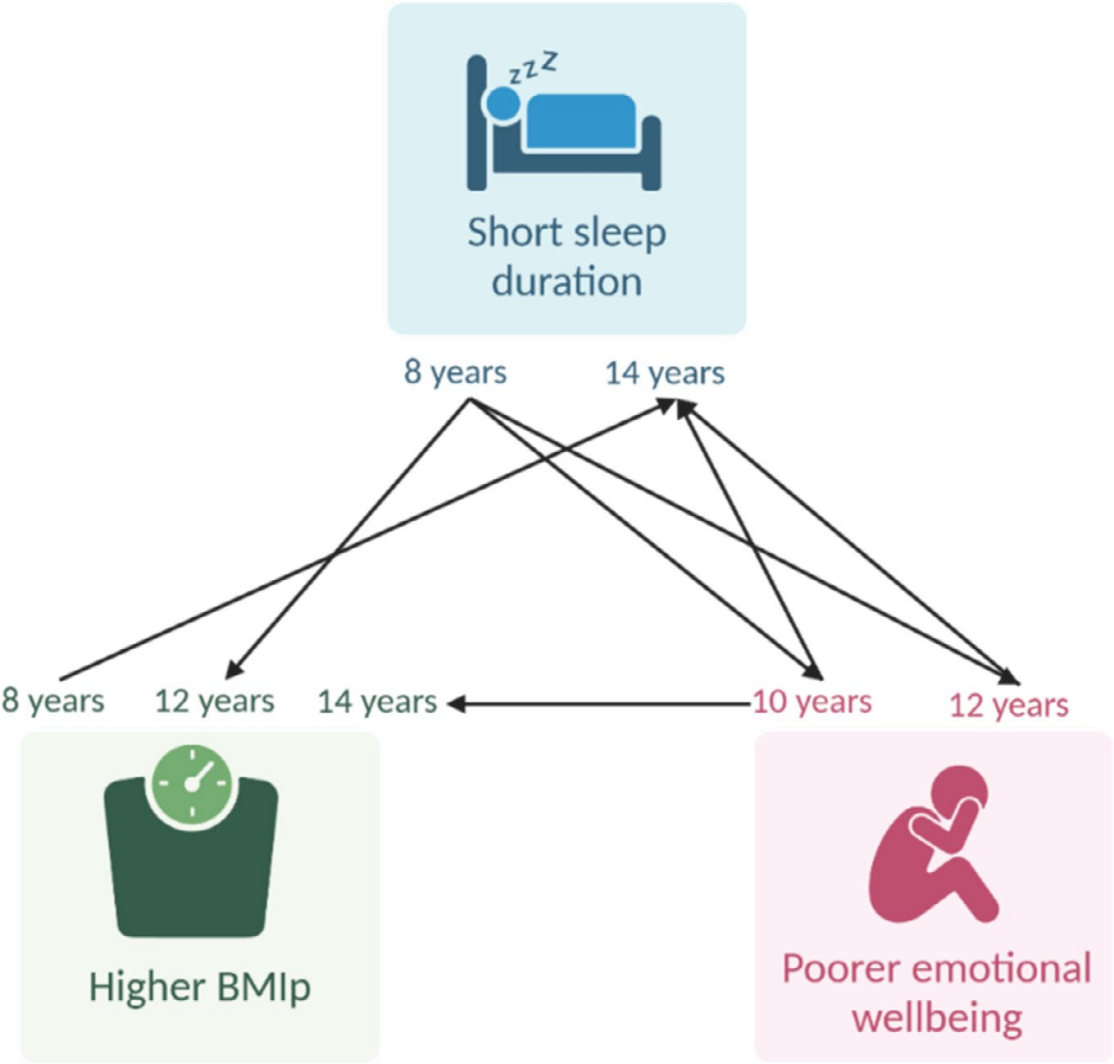
Overall findings of the bidirectional and longitudinal relationship of sleep, obesity and emotional wellbeing between 8 and 14 years old. BMIp, body mass index percentile.

The present study identified clear longitudinal and bidirectional associations between sleep, emotional wellbeing and BMI percentile from ages 8 to 14 (Figure 1). Shorter sleep duration at 8 years predicted both higher BMIp at 12 years and poorer emotional wellbeing at 10 and 12 years, while poorer emotional wellbeing at 10 and 12 years predicted shorter sleep at 14 years. In addition, higher BMIp at 8 years was associated with shorter sleep at 14 years, and poorer emotional wellbeing at 10 years was linked to higher BMIp at 14 years. Although not all hypothesised pathways were observed, the consistent pattern of associations demonstrates that these domains are interconnected across late childhood and adolescence. Effect sizes ranged from *β* = −0.07 to −0.19 for negative associations and up to *β* = 0.14 for positive associations, indicating modest but meaningful relationships. Taken together, these findings highlight a developmental interplay between sleep, emotional wellbeing and BMIp that unfolds across this critical period, suggesting that interventions which address these factors together may be most effective.

### 4.2 Longitudinal Relationship Between Sleep and Obesity

The findings from the current analyses suggest that a higher BMIp at 8 years was significantly associated with a shorter sleep duration at 14 years and reciprocally, a shorter sleep duration at 8 years was associated with a higher BMIp at 12 years, indicating a bidirectional relationship between sleep and obesity across puberty. This bidirectional relationship between insufficient sleep and obesity has been observed in later childhood in previous research that analysed two large longitudinal datasets (GINIplus and LISA studies) (Wang et al. 2023). Wang et al. (2023) identified that poor sleep in adolescence can contribute to an increased risk of overweight and obesity in early adulthood, and that being overweight or obese in adolescence can contribute to insufficient sleep in early adulthood. However, few comparisons to other data can be drawn on the age group that we have analysed in this study, due to limited existing longitudinal and bidirectional analyses. This is typically due to studies examining sleep at only one time point and obesity at multiple time points, demonstrating causality in one direction (Bagley et al. 2015; Fan et al. 2018; LeMay‐Russell et al. 2021; Lim et al. 2019; Lin et al. 2020; Michels et al. 2014; Snell et al. 2007).

In addition to insufficient sleep, oversleeping on a weekend and insomnia symptoms have been shown to increase in the general population during puberty in comparison with pre‐puberty (Fernandez‐Mendoza et al. 2021), indicating that the root cause of short sleep might involve underlying insomnia and delayed sleep onset. Duraccio et al. (2022) investigated the connection between weight status and insomnia severity in a clinically referred paediatric sample, revealing that higher levels of overweight or obesity were associated with more severe insomnia symptoms.

### 4.3 Longitudinal Relationship Between Sleep and Emotional Wellbeing

The current findings indicated a longitudinal and bidirectional relationship between shorter sleep and poorer emotional wellbeing: specifically, that short sleep duration at 8 years old was significantly associated with poorer emotional wellbeing at 10 and 12 years old, and that poorer emotional wellbeing at 10 and 12 years old was associated with shorter sleep duration at 14 years old. Existing literature supports the notion that short sleep duration or restriction of sleep can cause both emotional and physical wellbeing decline acutely and chronically, and that in turn, poor emotional wellbeing can cause shorter and more disturbed sleep patterns (Bacaro et al. 2024; Blunden et al. 2019; Bernert et al. 2017; Bei et al. 2016; Robillard et al. 2015; Vanderlind et al. 2014; Haack and Mullington 2005). A study in adults found that 50% sleep restriction over 12 days led to a 15% decline in optimism and sociability, highlighting the impact of cumulative sleep loss on emotional wellbeing (Haack and Mullington 2005). In adolescents and young people, day‐to‐day variability in sleep–wake patterns has been linked to mental health outcomes: delayed and irregular sleep onset times were more frequent in those with diagnosed mental health disorders (Robillard et al. 2015), short sleep and insomnia symptoms predicted acute depressive symptoms (Vanderlind et al. 2014), and increased variability in sleep timing, insomnia and nightmares were associated with acute suicidal ideation (Bernert et al. 2017). Conversely, Blunden et al. (2019) observed that over 81 days, depressive, anxiety and stress symptoms predicted temporal changes in sleep patterns amongst 4–17‐year‐olds. Bacaro et al. (2024) conducted a meta‐analysis that found a bidirectional relationship between poor sleep (including short sleep, low sleep quality and insomnia symptoms) and poorer mental health, characterised by increased internalising symptoms. Prolonged impaired and fragmented sleep, along with poor diet and reduced physical activity, has been found to increase immune inflammation (Haapala et al. 2022), induce oxidative and nitrosative stress (Hertiš Petek et al. 2022), reduce neuronal plasticity (Mandolesi et al. 2017) and neurotransmitter imbalances (Sejbuk et al. 2022) and contribute to the development of depression and other mental health disorders (Lopresti et al. 2013; Meerlo et al. 2015).

### 4.4 Longitudinal Relationship Between BMIp and Emotional Wellbeing

The current analyses identified a longitudinal relationship between poorer emotional symptoms at 10 years and a higher BMIp at 14 years, but not vice versa. In a previous analysis of the GUS dataset, it was identified that emotional wellbeing started to decline significantly around the age of 10 years (Gale, Cecil, and Williams 2024), indicating that this could be a trigger point for subsequent poor health, as observed in the current analyses. Obesity has also been reported to be most prevalent to develop around the age of 11–14 years (Howe et al. 2015) and this might indicate that poorer emotional wellbeing could be a trigger or indicator of a higher risk of the development of obesity.

In support of the current findings, a systematic review and meta‐analyses of longitudinal studies on the relationship between obesity and depression over a 20‐year period identified that having depressive symptoms in adolescence increased the individual's risk of developing subsequent obesity in later adolescence and early adulthood (Luppino et al. 2010). Additionally, overweight and obesity increased the risk of subsequent onset of depression in adults, but not when the obesity was present in adolescence (Luppino et al. 2010). These studies highlight that longitudinally, poorer emotional symptoms in adolescence may contribute to the subsequent development of obesity but the bidirectional nature of the relationship between a higher BMIp and emotional wellbeing occurs later in life. These findings are supported by mechanistic research that states, increased cortisol and insulin imbalance as a result of an increase in stress and anxiety can contribute to increased ghrelin and decreased leptin, which can in turn lead to weight gain and the development of obesity and type 2 diabetes (Hirotsu et al. 2015). Thus, poor wellbeing could be considered a cause or potential risk of the development of obesity.

### 4.5 Emotional Wellbeing as a Potential Mediator Between Sleep and Obesity

Previous research supports the idea that emotional wellbeing may mediate the relationship between poor sleep and obesity (Saleh‐Ghadimi et al. 2019; Gomes et al. 2019; Magee et al. 2010). Conversely, some studies propose that poor sleep could mediate the association between wellbeing and obesity (Song et al. 2022; Yu et al. 2016; Senna et al. 2013), although this remains unclear based on the current data. The current findings, along with prior studies, highlight the importance of exploring emotional wellbeing as a potential mediator. Wellbeing may either strengthen the connection between sleep and obesity or influence other pathways leading to the development of poor sleep and obesity in adolescents.

### 4.6 Strengths and Limitations

A major strength of this study is the use of the GUS dataset, the only longitudinal birth cohort dataset in Scotland that allows for the analyses of bidirectional relationships between sleep, obesity and emotional wellbeing over childhood and adolescence. The dataset includes a large sample of over 5000 participants in the original cohort, providing robust data for longitudinal analyses. Another key strength is the breadth of health‐related questions and objective measures, which allowed for comprehensive analyses across multiple domains, including obesity, sleep and emotional wellbeing and allowing for the adjustments of both child and caregiver demographics in the regression analyses. These outcomes were recorded fairly consistently across sweeps, enabling detailed and regularly measured intervals for bidirectional analyses from childhood to adolescence.

This study had several limitations. Participant attendance was inconsistent across sweeps, with many individuals from earlier and mid‐sweeps not participating in sweep 10, particularly during the COVID‐19 pandemic. This resulted in variability in the number of valid records available for analyses and the presence of non‐random missing data. Consequently, the final sample used in this study may not be fully representative of the original GUS cohort or the wider Scottish adolescent population. While GUS aimed to maintain national representativeness, attrition over multiple sweeps may have introduced selection effects. This should be considered when interpreting the findings, as differences between those who remained in the study and those lost to follow‐up could influence the observed associations. Additionally, while obesity and emotional wellbeing were measured consistently, sleep outcomes were only assessed at the beginning (age 8) and end (age 14) of the study period, leaving a significant gap in data collection. This gap limited the ability to pinpoint when sleep deterioration had the greatest impact on wellbeing and obesity during this critical developmental period. Despite this, bidirectional relationships between sleep, obesity and emotional wellbeing were examined using data from the two available time points.

## 5 Conclusions

This study demonstrates that sleep, emotional wellbeing and BMIp are interconnected across late childhood and adolescence, with bidirectional relationships emerging from as early as age 8 years. These findings highlight the need for multi‐component programmes that target sleep, emotional wellbeing and lifestyle behaviours together, recognising their combined influence on health trajectories. In Scotland, priority settings for such work include education, where schools can embed sleep and wellbeing into existing health curricula alongside nutrition and physical activity; families and communities, where supportive environments can reinforce positive routines; and the National Health Service, where preventive strategies can be integrated into child health services. Positioning sleep and wellbeing as core pillars of health promotion within these settings will be key to disrupting cycles of poor sleep, declining wellbeing and obesity risk as children transition into adolescence.

## Author Contributions

**Emma Louise Gale:** conceptualisation, data curation, investigation, formal analysis, methodology, project administration, writing – original draft, writing – review and editing. **Joanne Elizabeth Cecil:** conceptualisation, methodology, supervision, writing – review and editing. **Andrew James Williams:** conceptualisation, data curation, formal analysis, methodology, supervision, writing – review and editing.

## Conflicts of Interest

The authors declare no conflicts of interest.

## Data Availability

The data that support the findings of this study are available from UK Data Service. Restrictions apply to the availability of these data, which were used under license for this study. Data are available from the author(s) with the permission of UK Data Service.
